# Acculturation and Its Effects on Health Risk Behaviors among Myanmar Migrant Workers: A Cross-Sectional Survey in Chiang Mai, Northern Thailand

**DOI:** 10.3390/ijerph17145108

**Published:** 2020-07-15

**Authors:** Thin Nyein Nyein Aung, Yoshihisa Shirayama, Saiyud Moolphate, Thaworn Lorga, Motoyuki Yuasa, Myo Nyein Aung

**Affiliations:** 1 Department of Public Health, Graduate School of Medicine, Juntendo University, Tokyo 113-8421, Japan; drthinnyeinaung@gmail.com (T.N.N.A.); moyuasa@juntendo.ac.jp (M.Y.); 2 Faculty of International Liberal Arts, Juntendo University, Tokyo 113-8421, Japan; shirayam@juntendo.ac.jp; 3 Department of Public Health, Faculty of Science and Technology, Chiang Mai Rajabhat University, Chiang Mai 50300, Thailand; saiyudmoolphate@gmail.com; 4 School of Nursing, Mae Fah Luang University, Chiang Rai 57000, Thailand; thaworn.lorga@gmail.com; 5 Advanced Research Institute for Health Sciences, Juntendo University, 2 Chome-1-1 Hongo, Bunkyo City, Tokyo 113-8421, Japan

**Keywords:** acculturation, health risk behaviors, Myanmar migrant workers, non-communicable diseases, Thailand

## Abstract

Thailand hosts many workers who have migrated from neighboring countries and is facing a large burden of non-communicable diseases (NCDs). Health screening for migrant workers routinely emphasizes infectious diseases but overlooks NCDs. We surveyed prevalent health behaviors for NCDs and their influencing factors, particularly cultural adaptation patterns among Myanmar migrant workers in Chiang Mai, Northern Thailand. A total of 414 migrant workers consented to participate in the study. Lack of exercise (75.8%), current alcohol consumption (40.8%), current smoking (26.9%), and central obesity (24.3%) were major lifestyle problems. Being female and uneducated was associated with a lack of exercise. Current alcohol consumption was significantly associated with being male and being of Myanmar ethnicity, with an integrative strategy for acculturation, and with a higher income. Male participants and participants with a lower mean score of marginalization were more likely to smoke. Central obesity was associated with being older than 40 years, being female, engaging in an assimilation strategy, and being uneducated. These findings highlight the need for gender inclusive health promotion, the screening of NCD risk behaviors, and timely health education for migrant workers. It may assist authorities to devise strategies to extend health promotion and universal health coverage to the migrant population.

## 1. Introduction

International migration is a global phenomenon, and migrant workers are recognized as the most vulnerable population in society [1]. Foreign workers are commonly employed in jobs involving heavy physical loads with dirty operating environments because of their language limitations and cultural differences with the host culture. They are often engaged in low skilled jobs known as “3 D jobs”—i.e., dirty, difficult (sometimes demanding or degrading), and dangerous. Most of these workers are daily wagers, and are employed in the agricultural sector, on construction sites, and in a domestic capacity. These migrants are at risk for health disparities relative to the nature of their jobs and legal status. Moreover, language and cultural differences are a relevant obstacle to effective safety training at the workplace. This phenomenon is very significant in EU countries, as well as in the US, especially for migrant workers in the construction sector and in agriculture [2,3,4]. Labor intensive occupations and language barriers might minimize such workers’ social interactions and have a negative impact on their patterns of acculturation. A lack of social cohesion between migrants and their host societies can lead to the exclusion of migrant communities, and their occupational health and safety conditions can have a large impact on their health and acculturation. Migrant workers are at an increased risk of being marginalized and exploited by their employers, having poor access to health services, and experiencing a low sense of community belonging, all of which can affect their mental and physical health [5]. The migration experience can have a direct negative impact on migrants’ risky behaviors, such as smoking, drinking alcohol, and substance abuse [6]. The impact of the acculturation process on migrant health can be diverse, depending on both individual and contextual characteristics. Immigration may have positive or negative impacts on the health of migrants [7]. They can experience a healthy immigration effect, where newly arrived migrants tend to be healthier than the host population due to a positive selection bias by the formal immigration process, or an immigrant paradox, where there is an apparently positive association with acculturation, but which involves losing one’s heritage culture and gaining health risks [8]. Acculturation is a lengthy and fluid process through which migrants adapt, adopt, and acquire the host culture. The four strategies of acculturation were defined by Berry based on two basic issues: an individual’s relative preference for maintaining his or her heritage culture and identity and his or her relative preference for participating in the host culture. An assimilation strategy occurs when individuals shed their heritage culture and become absorbed into the host society. In contrast, when individuals place value only on their heritage and seek to avoid interaction with others, they undergo a process of separation. The process is called integration when there is an interest in both maintaining one’s heritage culture and engaging in daily interactions with the host, while marginalization occurs when individuals are excluded from both their heritage culture and their host culture [9,10]. There are many studies of the health inequalities and criticism of how acculturation affects health risk behaviors among Asian migrants in western countries. However, such studies remain scarce in migration settings within Asian countries [11,12,13,14,15].

Labor migration is also an established feature in the South East Asia context, and Thailand is one of the four receiving countries for international migration in this region [16]. People from neighboring countries migrate to Thailand for job opportunities with different immigration statuses. Thailand is home to migrant workers from neighbors such as Myanmar, Cambodia, and Laos; about 80.0% of migrant workers in Thailand were from Myanmar as of 2016. There were approximately 3.9 million registered migrant workers in Thailand in 2018, and Myanmar migrant workers made up the largest proportion of about 2.3 million [17]. Chiang Mai is close to the Shan state of Myanmar and hosts 81,299 (Male 41,428: Female 39,871) Myanmar migrant workers [18]. The majority of these workers are ethnically Myanmar or Shan, who share more-or-less similar cultural and religious practices. However, their Thai language skills and cultural adaptation may be different. Their acquired lifestyle changes and epidemiological risk factors have become interesting issues in public health. The Thailand migration report 2019 noted that the incidence of noncommunicable diseases (NCDs) in the migrant population was poorly known but remained the leading cause of death among Thais [16]. NCDs can make migrants vulnerable as such diseases can require regular and lifelong treatment. Moreover, NCDs can affect jobs, reduce life expectancy, and create a burden on the host countries [19].

Currently, health screening for migrant workers in Thailand includes mainly infectious diseases, such as tuberculosis and sexually transmitted diseases. Neither common NCDs nor lifestyle related risk behaviors are screened regularly. NCDs accounted for 74.0% of total deaths in 2016 and were predicted to continue to increase rapidly [20]. NCDs, especially cardiovascular diseases (CVDs), stroke, diabetes, cancer, and chronic lung diseases, are predominant killers in Thailand. Behavioral risk factors for CVDs (tobacco use, insufficient physical activity, harmful use of alcohol, and an unhealthy diet) and metabolic risk factors (increased blood pressure, overweight/obesity, increased cholesterol, and blood sugar) are highly prevalent in the Thai population [21]. According to WHO Thailand, about 40.0% of Thai adults smoke, and tobacco related deaths constitute almost one fourth of all CVD deaths. Moreover, the CVDs among younger people are more likely to be caused by tobacco smoking [22]. Globally, in 2016, alcohol caused 3.3% of all CVD deaths and 7.2% of all premature deaths. The current per capita alcohol consumption of 8 L in Thailand is the highest in the WHO South East Asian region. Drinking alcohol is associated with risks of developing health problems such as mental and behavioral disorders, major NCDs like CVDs, liver cirrhosis and cancer, and injuries [23]. Obesity is associated with an increased incidence of various CVDs and may also be an independent risk factor for CVDs [24]. According to WHO Thailand, about 31.6% of Thai individuals were overweight, and 9.2% were obese in 2016. In addition, Thailand ranks second in ASEAN in terms of its prevalence of obesity after Malaysia. About 14.6% of Thai people are physically inactive, and insufficient physical activity is a key risk factor for NCDs. Participation in sports and exercise activities is associated with a reduction in the risk of NCDs and improved mental health and general wellbeing [25,26]. While regular participation in exercise has many positive outcomes, little is known about how acculturation affects participation in physical activity or impacts health.

Myanmar migrant acculturation patterns in Chiangmai, as noted in both qualitative and quantitative studies, are mostly those of separation and integration, followed by assimilation and marginalization [27,28,29]. CVD risk factors and their associations with acculturation have been studied in the United States and Australia [30,31]. Previous studies reported the health risks of Myanmar migrants in Thailand but how such cultural adaptation influenced migrant health has not been studied yet in the context of northern Thailand [29,32,33,34]. The determination of modifiable risk factors and other associated factors may reduce the NCD burden on the host country. This study aims to elucidate the health behaviors of migrant workers and how acculturation affects those and impacts migrant health. The results might provide useful hints for launching a regular program for screening lifestyle related risk behaviors for NCDs and their prevention. Migrants are likely to experience specific health challenges, and the social determinants of health are complex and interrelated. Research focusing on the health and social security threats of migrant populations remains necessary to ensure a healthy global workforce.

## 2. Materials and Methods

### 2.1. Data Collection and Participants

This study was conducted in accordance with the Declaration of Helsinki [35]. The Ethical Review Committee for Research in Human Subjects: Boromarajonani College of Nursing Nakhon Lampang: Praboromarajchnok, Institute for Health Workforce Development, Ministry of Public Health, Thailand (approval number E 2560/39, dated October 31, 2017) approved the ethics of the study. The director of Provincial Employment Office, Chiangmai, Thailand (approval number 533.02.09, dated November 22, 2017) issued the official letter to recruit the migrant workers. The sample was calculated using the Taro Yamane formula with a 95% confidence level and the formula used was stated as *n* = [N/1 + N(e)2], where *n* = sample size required; *n* = total population; and e = tolerable error (0.05 or 95%) [36]. The population of 81,299 Myanmar migrants working in Chiang Mai province was considered as the total population and a sample size of 398 was calculated. After adding 5% for incomplete information, a total of 414 participants were included in the final analysis. Data collection was done in December 2017 at the Chiangmai provincial employment office where there is a one stop service for the registration of migrant workers in collaboration between the Thailand and Myanmar governments. Data were collected via self-administered or interviewer-administered survey questionnaires. The fifteen research assistants were public health students fluent in the languages of the study participants: Shan, Myanmar, and Thai. Using a stratified sampling technique, a total of 414 migrants were recruited according to their queue numbers in a Shan: Myanmar ratio of 3:1, with even numbers recruited on one day and odd numbers on the other days. The survey instrument and consent cover letter were available in both the Myanmar and Thai languages. Adult migrants (18 to 60 years old) who were legally working in Thailand and willing to participate in our study were included with their written informed consent, and unconsenting migrants were excluded. Participants received a small gift (around 1 US dollar in value, e.g., a coffee mug) after completing the survey.

### 2.2. Measures

Dependent variables included health-related behaviors: current smoking, current alcohol consumption, a lack of exercise, and central obesity.

Smoking: For smoking status, we created a dichotomous variable to capture the self-reported current smoking status, defining current smokers as those who smoke any tobacco products (cigarettes, tobacco, or cheroot) either on some days or every day. Individuals categorized as current non-smokers included respondents who were former smokers or those who never smoked cigarettes.

Alcohol: Drinking alcohol was defined as the consumption of any type of alcohol (spirit, beer, or wine), and consumption was measured as days of drinking per week and types of alcohol. A dichotomous variable was created for current alcohol drinkers and nondrinkers (for those who were ex-drinkers or teetotalers).

Exercise: The number of exercise sessions per week for any type of exercise (walking, running, playing football, or badminton) was assessed. A dichotomous scale of “lack of exercise”, for those who never exercise, and “exercise”, for those who exercise at least once a week to two to three times a week, was recoded.

Central obesity: Waist circumference and hip circumference were measured by a standard measuring tape to the nearest 0.1 cm. The waist: hip ratio was then calculated, and a value of greater than or equal to 0.9 for males and 0.85 for females was regarded as central obesity according to the WHO cutoff points; less than 0.9 for male and 0.85 for female participants was considered no central obesity [37].

Independent variables included demographic characteristics, socioeconomic status, and acculturation.

Demographic variables: These variables included age, grouped into the following categories: age (younger than 40 years of age or 40 years and above), gender (male or female), and race (Shan or Myanmar).

Socioeconomic status: Education and estimated monthly income were measured to assess the socioeconomic status of the participants. Educational status was assessed based on a list of six preset responses: formal education, primary school, middle school, high school, vocational college, or university. Education was recoded into a dichotomous scale including the no formal education group and those with primary school education and above. The minimum lowest wage for migrant workers in Thailand was about 300 Thai baht per day, and the participants were divided into two groups depending on an estimated monthly income of less than 9000 baht and an income greater than or equal to 9000 baht.

Acculturation: A literature review was conducted to identify the East Asian Acculturation Measure (EAAM) scale as a validated scale, which was adapted for Myanmar migrants in Thailand. The WHO recommended transcultural translation of the instrument was followed, and the overall Cronbach’s alpha reliability coefficient of the EAAM was 0.73 for the Myanmar version and 0.82 for the Thai version [38]. We used a 7-point Likert scale ranging from 1 (strongly disagree) to 7 (strongly agree), and the relevant subscale items were summed and divided by the number of items to yield a mean score for each strategy. The scores were then recoded as dichotomous variables, with 0 = a score less than 3.50 and 1 = a score greater than or equal to 3.50, in accordance with Barry (2001) [39].

### 2.3. Data Analysis

We used IBM SPSS version 22 (IBM Corporation, Armonk, NY, USA) for data analysis. Initially, the data were cleaned, and an exploratory analysis was conducted (including recoding the variables and computing the subscales and scales as needed). Demographic characteristics and socioeconomic status variables were analyzed by a descriptive analysis. Frequency and percentage were used for the categorical variables (age group, gender, education level, ethnicity, and estimated monthly income), and the mean (M) and standard deviation (SD) were used for continuous variables (the duration of stay in Thailand). Health risk behaviors, current smoking, current alcohol drinking, a lack of exercise, and central obesity were the dependent variables. Binary Logistic regression was used to identify the independent effects of the demographic factors, socioeconomic status, and acculturation on each health risk behavior. Adjusted odds ratios (adj OR) with a 95% confidence interval and a *p* value of <0.05 were considered to be significantly associated factors.

## 3. Results

### 3.1. Sample Characteristics

The mean age of the study participants was about 29.45 ± 9.03 years, with males constituting 55.8% of the group. Most of the participants were first-generation migrants (99.5%) of Shan ethnicity (87.0%), and their mean duration of stay in Thailand was 6.36 ± 5.70 years. About 49.0% of the participants did not have any formal education, and 64.6% earned an estimated monthly income of greater than or equal to 9000 baht. The characteristics of the study participants are described in Table 1.

### 3.2. Acculturation

**Figure 1:**
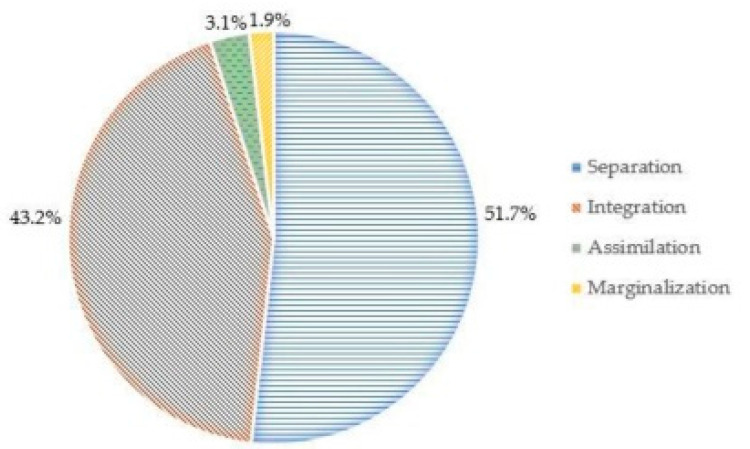
Acculturation strategies of the study participants, Myanmar migrant workers, in Chiang Mai, Thailand, 2017, (*n* = 414).

According to the findings of the EAAM, the mean (M) of the overall four strategies of acculturation was 3.75, and the standard deviation (SD) was 0.58. For the acculturation processes of Myanmar migrant workers, separation ranked the highest (M = 4.94, SD = 1.15), integration ranked second (M = 4.56, SD = 1.35), assimilation ranked third (M = 2.96, SD = 1.18), and marginalization ranked fourth (M = 2.53, SD = 1.12). The frequency of each acculturation strategy of the study participants is shown in Figure 1.

Pearson correlation was used to analyze the correlation between the four acculturation strategies. The correlation coefficient was set to r, and the significance level *p* was set to 0.05. The results are shown in Table 2. There was a significant positive correlation between integration and assimilation (r = 0.45, *p* = 0.00). However, separation had significant negative correlations with both assimilation (r = −0.46, *p*= 0.00) and integration (r = −0.23 *p* = 0.00). The marginalization strategy had a significantly positive correlation with assimilation (r= −0.23, *p* = 0.00), but a significantly negative correlation with integration (r= −0.13, *p* = 0.00). Therefore, the more likely the migrant workers were to be marginalized, the more likely they were to be assimilated, and the less likely they were to be integrated. There was no significant correlation between marginalization and separation (r = −0.04, *p* = 0.45).

### 3.3. Prevalence of Health Risk Behaviors

About 40.8% of the study participants were current alcohol drinkers, and spirits were the most common type of alcohol (50.9%), followed by beer (47.3%) and wine (1.8%). Among the participants, 26.3% were current smokers, with cigarettes representing the most common type of smoking. About 314 participants (75.8%) did not exercise, and 24.9% were centrally obese.

### 3.4. Health Risk Behaviors and Associated Factors

Health risk behaviors and significant associated factors are described in Table 3.

Alcohol: The male participants drank alcohol 21 times more often than female participants. Those with a Myanmar ethnicity, a higher monthly income, and an acculturation status categorized as “integrated” drank alcohol two times more frequently than others.

Smoking: Male participants and participants with a lower marginalization mean score were more likely to be current smokers. The most common type of smoking was cigarettes (65.4%), followed by hand rolled smokes (29.0%) and cheroots (4.7%).

Lack of Exercise: The female participants and those with no formal education did not do any exercise.

Central obesity: Being older than 40 years and female, having a higher assimilation score, and being uneducated were significantly associated with central obesity.

## 4. Discussion

The majority of the migrant workers from Myanmar display some risky health factors, such as current smoking, current alcohol consumption, a lack of exercise, and central obesity. The demographic factors affecting those health risk behaviors included old age, gender, and having Myanmar ethnicity. The socioeconomic factors of higher monthly income and poor education significantly affected the health risk behaviors of our study participants. Ageing is a major risk factor for CVDs, not only because of vascular ageing per se but also because ageing increases the time of exposure to other CVD risk factors [40]. Our study participants who were older than 40 years were significantly associated with central obesity. This finding is particularly alarming for the host country, which could be burdened by premature death and disability among ageing migrant workers. Just as men tend to smoke and drink more often than women in Myanmar culture, there were more male smokers and alcohol users in our study population, similar to other studies on Myanmar migrant workers in Thailand [41,42,43]. Nonetheless, a lack of exercise and central obesity were significantly more common among women. This finding, of women having more insufficient physical activity and greater obesity than men, is consistent with the 2016 Thailand national prevalence of physical inactivity (28.0% vs. 23.0% and 14.0% vs. 8.0% for obesity among men and women, respectively) [44].

Culture and the accessibility of exercise activities might influence the insufficient physical activity and obesity among female migrant workers. The health risk behaviors of alcohol and smoking were more common among male participants than females, and males were more physically active than females. Possibly, males prefer to be engaged in group activities featuring social interaction, such as playing team sports like football or socializing with friends and drinking. Race/ethnicity may affect disparities in the health indicators, reflecting the socioeconomic status and health outcomes of an individual. Even though only 13.0% of our study population were of Myanmar ethnicity, they tended to drink alcohol more frequently than the other ethnic group, Shan.

Education is one of the most important social determinants of health and encourages many positive health outcomes [45]. Educated individuals have better general and health related knowledge, overall health literacy and problem-solving skills, and access to health promotion services. However, nearly half of our study population (49.0%) were uneducated, which is similar to the results of another study in Chiang Rai, Northern Thailand, where 51.6% of the study participants never had any formal education [46]. The significant association between poor education and a lack of exercise and central obesity was also an important finding for CVD risks.

It is widely recognized that there are important links between economic resources and health that operate through diverse causal pathways. Both education and income can influence health outcomes, in part through pathways involving material resources. Most of our participants were daily wagers, and about 65.0% earned more than the estimated minimum monthly income of 9000 baht. This is a higher amount than that reported in another study in North East Thailand, where only 28.0% earned more than 9000 baht [5].

A statistically significant association between the study participants with higher monthly incomes and current alcohol consumption was noted in this study and is consistent with another study on Myanmar migrant workers in Chiang Rai, Northern Thailand [47]. A higher monthly income may have an impact on the accessibility and affordability of alcohol. Among the four patterns of acculturation, integration patterns are significantly associated with alcohol consumption. It is important for migrants to adapt to their new society’s culture and norms. However, a rich social network can be a double-edged sword. Alcohol is part of the culture of many Asian countries. Moreover, Thailand is ranked the highest for the consumption of alcohol among ASEAN countries. Well-to-do and socially integrated migrant workers may have more occasions to consume alcohol. A peer-based health promotion intervention program could be used to prevent excess alcohol consumption and its consequences among migrant workers in Thailand.

The effect of acculturation was complex, and we noted both positive and negative effects of acculturation on the health behaviors in this study population (integrated participants drank more alcohol and assimilated participants had a greater prevalence of central obesity). However, those with a lower marginalization score were significantly associated with current smoking. A separation strategy of acculturation did not have any significant association with health risk behaviors among the participants in this study. The participants with a higher integration mean score were associated with a two-times greater prevalence of current alcohol consumption than that of the lower mean score group. A similar finding was noted in another study on Myanmar migrant workers in Southern Thailand, which noted that the longer the duration of one’s stay in Thailand (a proxy measure for acculturation), the greater the chance of heavy alcohol consumption and alcohol related consequences because such migrants feel that they are free from migration related impacts and can adapt to the Thai context [34]. Greater acculturation and its relationship with alcohol consumption was also noted in a study of Asian American adolescents [48]. The participants with higher assimilation mean scores were centrally obese twice as often as those with lower mean assimilation scores. Myanmar migrants might experience a transition of epidemiological risk factors as they move from a poor country to an upper-middle income country and adopt the obesogenic food and sedentary habits of the host Thai culture, which had the second highest prevalence of obesity among ASEAN countries in 2016. A similar finding, with greater cultural assimilation being related to higher CVD risks, was noted in a study of Latino and Latina individuals in the USA. The authors explained that when migrants absorb into their host culture, they abandon the culture of their original country by reflecting the new country’s health norms [49]. We observed marginalization to be a protective factor against smoking, whereas in a study on adolescences in the USA, marginalization was associated with substance abuse, such as the use of alcohol and drugs [50].

To the best of our knowledge, this is the first study to explore acculturation and how it effects health risk behaviors of Myanmar migrant workers in a Northern Thailand setting. The implication of this study is that, although the recorded health risk behaviors are similar to those of other migrant workers in different regions of Thailand, their acculturation strategies and their effects on migrant health are different, and there is a need to further explore this phenomenon. However, there are some limitations in our study that should be considered in future research. Due to ethical reasons, our study participants included only registered migrant workers from Northern Thailand, who might not represent the characteristics of all migrants from Myanmar working both legally and illegally in Thailand. Moreover, most migrants in Chiangmai are ethnically Shan due to the geographical proximity between the Southern Shan state of Myanmar and Northern Thailand. Thus, their acculturation strategies may be different from those of migrant workers in other regions of Thailand. The similarity between the Shan and Northern Thai dialects may affect the Thai language skills of the Myanmar migrants, which could have an impact on their patterns of acculturation. As acculturation is a lengthy and fluid process, acculturation strategies may change with the development of Thai language proficiency and the duration of stay in Thailand. This study was limited by its cross-sectional design, which made it unable to describe the dynamic nature of the acculturation process taking place over time.

## 5. Conclusions

The majority of Myanmar migrant workers in Chiang Mai engage in unhealthy behaviors, such as smoking and drinking alcohol, especially male workers. With obesity and a lack of exercise being more prevalent among females, there is a need for gender-inclusive health promotion interventions. Male workers should be targeted for educational interventions concerning alcohol consumption and smoking cessation, whereas female workers should be targeted for physical activity promotion. Health education has had a positive impact on the health outcomes among these workers. The low level of education among the majority of migrants can lead those workers acquiring risky behaviors which may damage the population’s health. This will increase health care costs and impair productivity. Therefore, using screening to identify persons at risk alongside early health promotion is crucial. While social interaction and integration might have positive effects on migrant health, they can also lead to negative health consequences, including alcohol consumption. Healthy social interaction should be promoted to help solve the health risk behaviors acquired by migrant workers through the process of integration. Longitudinal studies are required to identify how acculturation changes over generations and how it is associated with health risk behaviors. Thailand is world-renowned for its universal health coverage and community-based health promotion for its citizens. Extending these benefits to the migrant population is an important step for regional public health and will be advantageous to the ASEAN economic community. We expect that the findings in our study will help devise culturally tailored strategies to promote healthy behaviors and reinforce the positive effects of acculturation, while counteracting possible negative health outcomes.

## Figures and Tables

**Table 1:** Characteristics of the study participants.

	Total	Current Smoking	Current Alcohol Consumption	Central Obesity	Lack of Exercise
	*n* (%)	*n* (%)	*n* (%)	*n* (%)	*n* (%)
Age					
18–39 years	348 (84.1)	91 (26.1)	143 (41.1)	262 (75.3)	76 (21.9)
40–60 years	66 (15.9)	18 (27.3)	26 (39.4)	52 (78.8)	27 (40.9)
Gender					
Male	231 (55.8)	106 (45.9)	154 (66.7)	30 (13.0)	162 (70.1)
Female	183 (44.2)	3 (1.6)	15 (8.2)	73 (39.9)	152 (83.1)
Ethnicity					
Shan	360 (87.0)	90 (25.0)	138 (38.3)	94 (26.2)	276 (76.7)
Myanmar	54 (13.0)	19 (35.2)	31 (57.4)	9 (16.7)	38 (70.4)
Education Level					
Formal education	211 (51.0)	54 (25.6)	90 (42.7)	39 (8.6)	147 (69.7)
No formal education	203 (49.0)	55 (27.1)	79 (38.9)	64 (31.5)	167 (82.3)
Monthly Income					
<9000 baht	266 (64.6)	29 (19.9)	38 (26.0)	61 (22.9)	194 (72.9)
>9000 baht	146 (35.4)	79 (29.7)	130 (48.9)	42 (29.0)	118 (80.8)
Separation					
Low	47 (11.4)	11 (23.4)	22 (46.8)	18 (38.8)	28 (59.6)
High	367 (88.6)	98 (26.7)	147 (40.1)	85 (23.2)	286 (77.9)
Integration					
Low	84 (20.3)	20 (23.8)	30 (35.7)	18 (21.7)	67 (79.8)
High	330 (79.7)	89 (27.0)	139 (42.1)	85 (25.8)	247 (74.8)
Assimilation					
Low	284 (68.6)	71 (25.0)	106 (37.3)	63 (22.3)	228 (80.3)
High	130 (31.4)	30 (29.2)	63 (48.5)	40 (30.8)	86 (66.22)
Marginalization					
Low	326 (78.7)	95 (29.1)	138 (42.3)	84 (25.8)	48 (76.1)
High	88 (21.3)	14 (15.9)	31 (35.2)	19 (21.6)	66 (75.0)

**Table 2:** Correlation between each of the acculturation strategies.

Acculturation Strategy	Separation	Integration	Assimilation	Marginalization
Separation	1	−0.226 **	−0.459 **	−0.037
Integration	-	1	0.446 **	−0.130 **
Assimilation	-	-	1	0.223 **
Marginalization	-	-	-	1

** Correlation is significant at a 0.01 level (2-tailed).

**Table 3:** Health risk behaviors and associated factors.

	Current Smoking	Current Alcohol Consumption	Central Obesity	Lack of Exercise
	Adj OR (95%CI)	Adj OR (95%CI)	Adj OR (95%CI)	Adj OR (95%CI)
Age 40–60 years vs. 18–39 years (ref:)	0.94 (0.46–1.90)	1.21 (0.60–2.44)	2.62 * (1.40–4.91)	0.95 (0.48–1.88)
Gender				
Male vs. Female (ref:)	52.49 ** (16.03–71.85)	21.40 ** (11.56–39.61)	-	-
Female vs. Male (ref:)	-	-	5.31 ** (3.10–9.11)	1.94 * (1.17–3.23)
Ethnicity Myanmar vs. Shan (ref:)	1.49 (0.69–3.22)	2.35 * (1.04–5.27)	1.15 (0.48–2.78)	0.84 (0.41–1.74)
Education level No formal education vs. Formal education (ref:)	1.25 (0.72–2.16)	1.00 (0.59–1.69)	1.91 * (1.14–3.2)	1.89 * (1.15–3.10)
Monthly Income >9000 baht vs. <9000 baht (ref:)	0.90 (0.49–1.63)	1.75 * (1.01–3.03)	1.13 (6.67–1.91)	1.33 (0.79–2.25)
Separation High vs. Low (ref:)	1.50 (0.62–3.63)	0.81 (0.34–1.90)	0.59 (0.28–1.28)	1.80 (0.89–3.67)
Integration High vs. Low (ref:)	1.66 (0.79–3.52)	2.18 * (1.06–4.48)	0.77 (0.38–1.56)	0.59 (0.34–1.03)
Assimilation High vs. Low (ref:)	1.30 (0.68–2.48)	1.40 (0.75–2.61)	2.08 * (1.14–3.79)	1.00 (0.55–1.80)
Marginalization Low vs. High (ref:)	2.23 * (1.06–4.68)	1.18 (0.60–2.30)	1.44 (0.77–2.69)	1.00 (0.55–1.80)

Adj OR: Adjusted Odd ratio, 95%CI: 95% Confidence Interval, * *p* < 0.05, ** *p* < 0.01.
